# Resilience, Burnout and Mental Health in Nurses: A Latent Mediation Model

**DOI:** 10.3390/jcm13102769

**Published:** 2024-05-08

**Authors:** Iván Suazo Galdames, María del Mar Molero Jurado, Elena Fernández Martínez, María del Carmen Pérez-Fuentes, José Jesús Gázquez Linares

**Affiliations:** 1 Facultad de Ciencias de la Salud, Universidad Autónoma de Chile, Providencia 7500912, Chile; ivan.suazo@uautonoma.cl; 2 Department of Psychology, University of Almería, 04120 Almeria, Spain; mpf421@ual.es; 3 SALBIS Research Group, Faculty of Health Sciences, University of León, 24071 Leon, Spain; elena.fernandez@unileon.es; 4 Department of Psychology, Universidad Autónoma de Chile, Providencia 7500912, Chile; jlinares@ual.es

**Keywords:** burnout, resilience, nursing, latent mediation model, mental health, COVID-19

## Abstract

**Background/Objectives**: The burnout syndrome in nurses has been related to the development of mental health problems. On the contrary, resilience is related to adequately coping with stressful situations and better mental health. The objective was to analyze the relationship between resilience and mental health problems in nurses and estimate the proportion mediated by burnout in the association. **Methods**: In 2021, a total of 1165 Spanish nurses were selected through a stratified random sampling method. Participants anonymously filled in the Resilience Scale (RS-14), the Maslach Burnout Inventory Survey, and the General Health Questionnaire. To test the hypothesis proposed and explain the mediating effect of burnout empirically, structural equation modeling (SEM) was applied. A latent mediation model was computed. **Results**: Resilience was negatively related to burnout and mental health problems. The direct relationship between burnout and the latent health variable was positive. In addition, in view of the total effect of resilience on mental health problems and the magnitude of the indirect effect, we stated that the proportion of this effect mediated by burnout ranged from 0.486 to 0.870. **Conclusions**: This study reveals that fostering resilience in nurses directly and indirectly reduces burnout and improves their mental health. The implementation of resilience programs and supportive institutional policies is recommended to improve working conditions and the quality of patient care.

## 1. Introduction

Nursing, especially during the time of COVID-19, is considered a hard profession, not free of risk. The lack of materials and indispensable close contact during the treatment of patients infected by SARS-CoV-2 leaves healthcare professionals at greater risk of infection, especially nurses, who are the group that spends the most time and interacts most with the patient [1]. In Spain, 86,000 healthcare workers had been infected, with 63 dying from the disease before 20 February 2021, which represented about 4.4% of the total infected population [2].

During the COVID-19 pandemic, healthcare workers were exposed to a multitude of psychosocial pressures harmful to their mental health, as well as difficult coping situations such as workplace violence [3]. Previous studies have reported increased stress, anxiety, depression, and psychological distress during the pandemic [1,4,5,6]. Fear of contagion by COVID-19 during the pandemic seemed to worsen distress and was found to be associated with negative mental health [7] and social anxiety symptoms [8]. Along this line, Wańkowicz, Szylińska, and Rotter [9] reported that healthcare workers who were exposed to patients infected with SARS-CoV-2 in emergency rooms, infection units and intensive care had a much higher risk of feeling anxiety and depression symptoms than those who worked in other units.

At the present time, burnout, defined as “physical, emotional exhaustion and mental state resulting from prolonged participation in emotionally demanding job situations” [10], seems to be one of the immediate effects of the pandemic on healthcare professionals [11,12]. Burnout has three dimensions as follows: exhaustion, cynicism, and low professional efficacy [13]. This syndrome has already shown high levels in healthcare professionals, especially nurses [14,15], but they have been rising with the challenges and the precedent that SARS-CoV-2 has been posing for all health systems around the world [9].

These data are of concern since exhaustion can reduce the quality of care [16] and cause errors in health care. Experiencing exhaustion leads to job dissatisfaction, patient dissatisfaction, low quality of life, and low-quality care. Furthermore, exhaustion contributes to the problem of a shortage of nurses [17]. Quality of care in nursing is of the highest importance at the present time and is a clear determinant in the evolution and safety of the patients, which is also often affected by hospital noise [18]. A high percentage of nurses are first-line personnel; that is, they belong to a group of healthcare professionals who are in direct contact with the user. This means that their actions, as well as their decisions, have an immediate effect on patients and on their safety; hence, the importance of civil responsibility for clinical diagnosis [19] requires the implementation of innovative solutions in nursing care during crises [20,21].

This syndrome is associated with health problems facilitating the development of physical and mental disorders [15,19,22]. The relationship between the burnout and health of Spanish nurses has been explored in previous studies, where low levels of stress are related to better health [23]. However, not everyone exposed to very intense crisis situations suffers from mental health disorders, so it is indispensable to determine the resources that some people have that could be acting as protective factors to avoid mental health problems [24,25]. Resilience has been demonstrated to be an essential resource acting as a protective variable that people must have to confront overwhelming situations and come out fortified by them [19,22], particularly protecting against burnout [26].

Personal resilience is considered a dynamic process by which people can adjust positively to adversity and moderate potential harm [27]. It helps people adopt coping strategies, minimizes anxiety, and develop problem-solving skills [22].

Resilience in nurses has been described as a tool or skill that enables them to overcome adversity in the workplace [28], concentrating on developing or improving the ability [29] to modify, balance, and control unfavorable environments [27] and find solutions to challenges [30].

Resilience minimizes and protects against the negative effects related to stress, such as burnout [31,32], and is a crucial component of well-being and the physical and mental health of nurses [33,34]. West et al. [35] examined a sample of 5445 US doctors and concluded that resilience is inversely associated with symptoms of exhaustion. In a study on Spanish nurses, the results showed that mental health was negatively related to the three dimensions of burnout and positively to resilience, concluding that resilience is not only important for improving the mental health of nurses but also for amortizing and minimizing the negative consequences of job stress [36]. It has also been observed that people with high resilience show less burnout [37].

However, it is not clear whether resilience has a mediating or moderating role [38]. One study concluded that resilience seems to have a small mediating role in some psychological variables [39]. In another study, resilience had a significant moderating effect on the relationship between work and exhaustion [40]. However, Arrogante and Aparicio-Zaldívar [36] concluded that in an intensive care unit, resilience partially mediated the relationships between exhaustion and depersonalization and mental health and fully mediated between personal accomplishment and mental health. Yu, Raphael, Mackay, Smith, and King [41] concluded in their bibliographic review that the job demands of nurses, such as stress, burnout, posttraumatic stress syndrome, and workplace bullying, were negatively associated with resilience, where resilience was mediated by attenuating the effects of work demands [42,43]. The COVID-19 pandemic exacerbated stress and burnout among healthcare workers, which increases the relevance of studying these phenomena. This study highlights the need to better understand how resilience can act as a buffer against the negative effects of work-related stress in this professional group.

Studies have been performed during the pandemic that have identified the resilience of nurses as a good mechanism against stress, anxiety, and depression [5,44,45]. Those who showed the most resilience experienced less depression and exhaustion [7], as it acted as a protective factor against depression [46] partially mediating in the relationship between depression and burnout [47]. Resilience is identified as a significant factor in reducing both burnout and mental health problems. This is critical given that burnout affects the quality of care and the well-being of nurses, with implications that have been accentuated by the pandemic.

An analysis of factors influencing burnout, resilience, and the health condition of nurses is necessary to design interventions that improve the levels of these constructs in social healthcare organizations, thereby improving the quality of patient care. Resilience has been demonstrated as capable of improving with training, which would then increase nurses’ well-being [44,48]. In view of the above, resilience would seem to be a protective factor of nurses’ mental health during the COVID-19 pandemic, but it is not clear what its influence is on the exhaustion of nurses during this period of pandemic or its influence on their mental health.

The study investigates how resilience influences, directly and indirectly, through burnout, the mental health problems of nurses during the COVID-19 pandemic. The mediating role of burnout in the relationship between resilience and mental health problems in Spanish nurses was explored.

The direct effect refers to how resilience directly influences mental health in nurses. In this line, a study by Mealer et al. [49] examined how resilience was directly associated with improvements in the mental health of nurses working in intensive care units. Moreover, the indirect effect occurs when resilience influences mental health through one or more intermediate variables (in this case, burnout). For example, in the study by Ferreira and Gomes [50], the results show that resilience can function as an inhibitor of all dimensions of burnout.

Therefore, the objective of this study was to analyze the relationship between resilience and the presence of the mental health problems in nurses and estimate the proportion mediated by burnout in that association.

The following hypotheses were posed:

**H1.**  *Resilience is negatively associated with burnout and with the presence of mental health problems of nurses.*

**H2.**  *Burnout is associated positively with the mental health problems of nurses.*

**H3.**  *Burnout mediates the relationship between the resilience and mental health problems of nurses.*

Along this line, the practical aim of this study was to provide an empirical basis for the design of programs and interventions that foster resilience among nurses, which could improve their mental health and the quality of care they provide, especially in high-stress contexts such as those experienced during the pandemic.

## 2. Materials and Methods

### 2.1. Procedure

The research was conducted in 2021 with the aim of exploring the relationship between resilience and mental health problems in nurses mediated by burnout. For this purpose, a representative sample of Spanish nurses was selected using a stratified random sampling method, thus ensuring the distribution of the sample in different regions and hospital centers. Participants were identified through professional nursing registries and contacted through professional association platforms. They were invited to participate in the research through a link distributed on social media and emails, which provided a broad and accessible recruitment method. Data were collected using a self-report approach, with participants completing three standardized questionnaires. Surveys were administered electronically, ensuring the anonymity and confidentiality of responses. No interviews were conducted as this study relied exclusively on self-administered questionnaires.

Data were collected with a CAWI (Computer-Aided Web Interviewing) survey. Participation was voluntary, and before starting to answer the questionnaire, the first page provided information about the study and its purpose. The participants gave their informed consent by marking a box designated for the purpose, which then gave them access to the questionnaire. They were asked to respond truthfully, ensuring the anonymity of their answers. Control questions were inserted in the questionnaire to detect random or incongruent answers. Randomly inserted control questions in a questionnaire are items designed to check the attention, consistency, and sincerity of participants’ responses, which is especially useful when questionnaires are self-administered.

The original sample included 1165 Spanish nurses. Incongruent or random answers were detected by applying filters with control questions placed throughout the questionnaire, and these cases were eliminated from the database (specifically, CQ_1_ discarded 66 cases, CQ_2_ 59 cases, and CQ_3_ 27).

This study was approved by the University of Almería Bioethics Committee (Ref: UALBIO2020/032).

### 2.2. Instruments

The Spanish version of Wagnild’s [39] 14-item *Resilience Scale* (RS-14) by Sánchez-Teruel & Robles-Bello [51] was used. This instrument measures the degree of individual resilience, a positive personality characteristic that enables the individual to adapt to adverse situations. The RS-14 uses a 7-point Likert scale, where 1 means “strongly disagree” and 7 means “strongly agree”. The scale comprised the following two factors: personal competence (11 items) and acceptance of oneself and of life (3 items). The author of the original scale proposed the following levels of resilience: 14–30 = very low, 31–48 = low, 49–63 = normal, 64–81 = high, and 82–98 = very high. In this study, reliability was *ω* = 0.70 for acceptance of oneself and of life.

The *Maslach Burnout Inventory Survey* (MBI) [52], specifically the Spanish version for human service professionals (MBI-HSS) [53], was employed to evaluate burnout. This instrument consists of 22 items that are rated on a seven-point Likert-type scale (where 0 is “never” and 6 is “every day”). These items are distributed in the following three dimensions: emotional exhaustion, which refers to feelings of physical, mental, and emotional fatigue at work (“*I feel emotionally drained from my work*”); depersonalization, which refers to self-criticism and loss of interest in the job (“*I have been losing enthusiasm for my work*”); and personal accomplishment, understood as feelings of competence (“*I have accomplished many worthwhile things in my profession*”). The reliability indices were *ω* = 0.88 for the full scale, *ω* = 0.91 for the emotional exhaustion subscale, *ω* = 0.64 in depersonalization, and *ω* = 0.79 for personal accomplishment.

*General Health Questionnaire* (GHQ-28) [54]. The Spanish adaptation validated by Lobo et al. [55] was applied. It consists of 27 items with four answer choices and provides information on somatic symptoms, anxiety and insomnia, social dysfunction, and depression. The Likert-type scoring method was used to rate each answer choice from 0 to 3. Response options may vary according to the question, reflecting increasing levels of symptoms or problems from “better than usual” or “no more than usual” to “much more than usual” or “much less than usual”. The instrument’s reliability was *ω* = 0.94 for the complete scale, and for each of the subscales, it was as follows: somatic symptoms (*ω* = 0.88), anxiety and insomnia (*ω* = 0.91), social dysfunction (*ω* = 0.77), and depression (*ω* = 0.86).

### 2.3. Data Analysis

The SPSS version 24.0 statistical package for Windows (IBM Corp. released 2016, Armonk, NY, USA) [56] was used for data processing and analysis. The McDonald’s [57] omega coefficient was estimated to determine the reliability of the evaluation instruments used following the recommendations of Ventura-León and Caycho [58].

The correlation matrix of the study variables was presented with mean scores and standard deviations.

**Figure 1:**
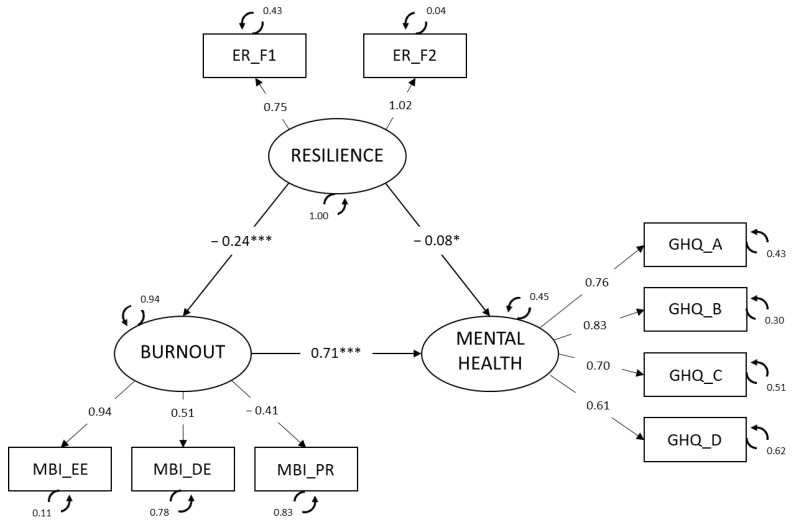
Structural equation model. Standardized parameters are shown. * *p* < 0.05, *** *p* < 0.001. (Note. Mental health = health measured with the GHQ-28, and therefore, interpreted negatively; that is, it corresponds to the presence of mental health problems. ER_F1 = Personal competence; ER_F2 = acceptance of self and life; MBI_EE = emotional exhaustion; MBI_DE = depersonalization; MBI_PR = personal accomplishment; GHQ_A = somatic symptoms; GHQ_B = anxiety/insomnia, GHQ_C = social dysfunction; and GHQ_D = depression).

To test the hypothesis proposed and explain the mediating effect of burnout empirically, an SEM was applied (Figure 1). A latent mediation model was computed using the *Diagonal Weighted Least Squares* (DWLS) method and specifying two paths of the impact of resilience (X) on the mental health of nurses (Y): a direct effect and an indirect effect through burnout (M). The lavaan package [59], in JASP version 0.14 [60], was used for this. The following indices were used to evaluate model fit: chi-square/degrees of freedom (χ^2^/*df*), which is considered optimum at <3 [61] and acceptable at <5 [62]; the CFI, TLI and GFI indices, which according to Hu and Bentler [63]) should be >0.95 to consider optimum fit and >0.90 for acceptable fit; and other indices, such as the RMSEA, which with an optimum at <0.06 and at <0.08 or very close, is considered acceptable.

## 3. Results

The final study sample was *n* = 1013. The mean age of the participants was 32.71 years (SD = 9.35), and 88.05% (*n* = 892) were women. Marital status was 56.66% (*n* = 574) married or with a stable partner, followed by singles who represented 38.89% (*n* = 394) of the sample, and a minority who were divorced/separated (4.24%, *n* = 43) or widowed (0.19%, *n* = 2).

### 3.1. Preliminary Analyses

Table 1 shows the correlation matrix of the study variables. As can be observed, resilience was negatively associated with emotional exhaustion and depersonalization and correlated positively with personal accomplishment. Resilience correlated negatively with all the GHQ-28 scale’s dimensions as follows: somatic symptoms, anxiety/insomnia, social dysfunction, and depression.

### 3.2. Structural Equation Model: A Latent Mediation Model

The hypothesized model (Figure 1) showed an acceptable fit, as shown by the following fit indices: χ^2^ (24) = 183.13, χ^2^/*df* = 7.63, *p* < 0.001, CFI = 0.96, TLI = 0.94, GFI = 0.96, and RMSEA = 0.081 (CI 90% = 0.070, 0.092). The relationships found between the latent variables in the model were as follows: resilience, which was negatively related to burnout (−0.24, *p* < 0.001), and the presence of mental health problems (−0.08, *p* < 0.05). The direct relationship between burnout and the latent health variable (constructed from the GHQ-28 dimensions) was positive (0.71, *p* < 0.001).

Moreover, in view of the total effect of resilience on mental health problems (0.026, *p* < 0.001) and considering the magnitude of this indirect effect (−0.17, *p* < 0.001), we can conclude that the proportion (indirect/total) of this effect mediated by burnout ranged from 0.486 to 0.870.

## 4. Discussion

The objective of this study was to analyze the potential mediating role of burnout in the association between the resilience and mental health problems of Spanish nurses during the COVID-19 pandemic.

Burnout generates mental health problems in nurses [15,22], and the pressure of care, along with other psychosocial factors derived from the COVID-19 pandemic, has worsened this problem, becoming a phenomenon experienced worldwide [1,4,5,12]. This study also showed that burnout is associated with mental health problems. The dimensions of exhaustion and depersonalization were positively correlated with the four scales on the GHQ-28 Health questionnaire, although the exhaustion scale showed a stronger correlation, especially with the somatic symptoms and anxiety/insomnia scales. The personal accomplishment dimension was also correlated, although negatively and weaker, with the four health scales, where the strongest correlations were social dysfunction and depression. These data were also confirmed in the structural equation model presented, where the direct relationship between burnout and the latent variable of health was positive. These results agree with previous studies performed with healthcare professionals [7,36,46].

The relationships found in this study between resilience and the three burnout dimensions, where the strongest correlation was between the accomplishment dimension and resilience, are also congruent with empirical evidence and confirm that resilience is associated with low levels of burnout and prevents the appearance of burnout syndrome in nurses [7,32,36,46]. In the study by Guo [32], the correlation indices of these variables were lower than in our study. The systematic review by Yu et al. [41] also showed that higher resilience in nurses could help reduce burnout and increase job commitment, improving their care practice when confronted with challenges in the workplace. This could help nurses form strategies for dealing with adversity and attenuate the effects of job demands. The bibliographic review by Foster et al. [43] on the mental health of nurses arrived at the same conclusion as did the meta-analysis conducted by Deldar et al. [42]. In the study developed by Ertem et al. [37], resilience was also observed to be related to burnout, and furthermore, the levels of resilience of nurses were influenced by factors such as age, sex, the unit they worked in, and job satisfaction, while burnout level varied with such factors as the unit worked in, work schedule, night shift and level of job satisfaction. However, it is influenced not only by job factors but also by individual and social characteristics [25].

Resilience has a significant moderating effect on the relationship between emotional work and exhaustion [40], as reflected by the negative relationship between resilience and burnout in the structural equation model.

The relationships between resilience and mental health also confirmed the results found by Arrogante and Aparicio-Zaldívar [36], Luceño-Moreno et al. [7], and Yöruk and Güler [46], who identified that resilience is associated with the healthier psychological profile of nurses. This study further contributes another important finding as follows: the significant mediating role of burnout in the relationship between resilience and nurses’ mental health. Resilience has a direct effect on nurses’ mental health and reduces the impact of negative consequences of burnout syndrome on mental health due to its indirect effect on itself. In the study by Guo et al. [32], resilience was already shown to be one of the main predictors of burnout, although in this relationship, other variables also came into play. This was also confirmed in the study by Kim et al. [5] with nurses during the COVID-19 pandemic.

All the findings of this study contribute to understanding the relationship between resilience, burnout, and mental health and verify that resilience is a tool for fighting exhaustion in nurses [34,47], for which there are already intervention programs [44,48].

Our study had some limitations. It was based on an interview spread over social networks, which could have been affected by self-selection bias, as the nurses who answered the survey might have been those with digital literacy or who were more aware of health problems. Due to this study’s cross-sectional design, interpretation of the mediation results must be taken with caution. It was carried out during a specific period during the pandemic, which meant that a longitudinal design was necessary to examine the long-term effects of the pandemic on nurses. There may also be other variables affecting the relationships between resilience, burnout, and mental health. In this sense, it would be useful to consider other elements in future research.

These results could lead to future lines of research, such as enlarging the sample size to include other healthcare professionals for a wider view of all the workers in the healthcare system, such as physicians, emergency medical technicians, and hospital support workers, who also face high levels of stress and burnout in their daily practice.

It might also be of interest to include students in these disciplines to see how the levels of these variables change from the beginning of their training. Finally, longitudinal studies are necessary to find out the changes that occur in the measurements made and analyze the dynamic nature of these constructs. Based on the results reported, an intervention strategy could be designed to reduce the wear on nurses and increase their levels of resilience, thereby reducing mental health problems, which could have repercussions for better quality of care.

## 5. Conclusions

In this study, we analyzed the health, burnout, and resilience of Spanish nurses and the relationships between these constructs during a period of the COVID-19 pandemic.

Based on these results, resilience can benefit the mental health of nurses directly and also indirectly by lowering burnout levels. This construct is fundamental for keeping nurses from developing mental health problems. Therefore, programs that are designed to improve mental health should include interventions that increase resilience. These programs should include stress management workshops, self-care techniques, and resilience skills development strategies, which are tailored to the needs and work realities nurses face on a daily basis.

These conclusions are significant for promoting the mental health of nurses and the design of prevention programs. Since resilience also influences the quality of care, our results should be kept in mind for the design of programs that improve nursing quality and patient safety. Because of the increase in care pressure due to the wave of infection, increasing the workload of nurses in a context of doubt and insecurity, burnout, and mental health problems could worsen. The solution requires more funding for the prevention and promotion of mental health. Thus, to improve working conditions for nurses, we recommend that healthcare institutions implement supportive policies that include adequate break times, accessible psychological support resources, and an equitable distribution of workload. These policies not only foster a healthy work environment but could also enable nurses to more effectively utilize the resilience resources provided, optimizing their well-being and professional effectiveness.

## Figures and Tables

**Table 1:** Resilience, burnout and health. Descriptive statistics and Pearson correlation matrix.

			Resilience M = 79.74, SD = 13.05		Burnout (MBI)	
				Exhaustion	Depersonalization	Accomplishment
Burnout (MBI)	Exhaustion	Pearson’s r	−0.121 ***	—		
	M = 29.76	Upper 95% CI	−0.060	—		
	SD = 12.33	Lower 95% CI	−0.182	—		
	Depersonalization	Pearson’s r	−0.114 ***	0.495 ***	—	
	M = 9.56	Upper 95% CI	−0.053	0.540	—	
	SD = 6.04	Lower 95% CI	−0.175	0.447	—	
	Accomplishment	Pearson’s r	0.439 ***	−0.304 ***	−0.303 ***	—
	M = 37.57	Upper 95% CI	0.488	−0.247	−0.246	—
	SD = 6.27	Lower 95% CI	0.388	−0.359	−0.358	—
Mental Health (GHQ-28)	Somatic symptoms	Pearson’s r	−0.090 **	0.571 ***	0.239 ***	−0.162 ***
	M = 10.83	Upper 95% CI	−0.028	0.611	0.296	−0.102
	SD = 5.20	Lower 95% CI	−0.150	0.528	0.180	−0.222
	Anxiety/insomnia	Pearson’s r	−0.122 ***	0.617 ***	0.285 ***	−0.166 ***
	M = 10.73	Upper 95% CI	−0.061	0.654	0.340	−0.105
	SD = 5.59	Lower 95% CI	−0.182	0.578	0.227	−0.225
	Social dysfunction	Pearson’s r	−0.195 ***	0.486 ***	0.240 ***	−0.267 ***
	M = 8.70	Upper 95% CI	−0.135	0.531	0.297	−0.209
	SD = 3.06	Lower 95% CI	−0.254	0.437	0.181	−0.324
	Depression	Pearson’s r	−0.289 ***	0.402 ***	0.242 ***	−0.255 ***
	M = 2.40	Upper 95% CI	−0.231	0.452	0.300	−0.196
	SD = 3.61	Lower 95% CI	−0.344	0.349	0.184	−0.312

Note. ** *p* < 0.01, *** *p* < 0.001.

## Data Availability

The data presented in this study are available on request from the corresponding author.
